# Levels of serum biomarkers from a two-year multicentre trial are associated with treatment response on knee osteoarthritis cartilage loss as assessed by magnetic resonance imaging: an exploratory study

**DOI:** 10.1186/s13075-017-1377-y

**Published:** 2017-07-20

**Authors:** Johanne Martel-Pelletier, Jean-Pierre Raynauld, François Mineau, François Abram, Patrice Paiement, Philippe Delorme, Jean-Pierre Pelletier

**Affiliations:** 1 Osteoarthritis Research Unit, University of Montreal Hospital Research Centre (CRCHUM), 900 Saint-Denis, Suite R11.412, Montreal, Quebec H2X 0A9 Canada; 2 Medical Imaging Research & Development, ArthroLab Inc, Montreal, Quebec Canada; 3 ArthroLab Inc, Montreal, Quebec Canada

**Keywords:** Knee, Osteoarthritis, Biomarkers, Clinical trial

## Abstract

**Background:**

There is an obvious need to identify biomarkers that could predict patient response to an osteoarthritis (OA) treatment. This post hoc study explored in a 2-year randomized controlled trial in patients with knee OA, the likelihood of some serum biomarkers to be associated with a better response to chondroitin sulfate in reducing cartilage volume loss.

**Methods:**

Eight biomarkers were studied: hyaluronic acid (HA), C reactive protein (CRP), adipsin, leptin, N-terminal propeptide of collagen IIα (PIIANP), C-terminal crosslinked telopeptide of type I collagen (CTX-1), matrix metalloproteinase-1 (MMP-1), and MMP-3. Patients were treated with chondroitin sulfate (1200 mg/day; *n* = 57) or celecoxib (200 mg/day; *n* = 62). Serum biomarkers were measured at baseline. The cartilage volume at baseline and its loss at 2 years were assessed by quantitative magnetic resonance imaging (MRI). Statistical analysis included analysis of covariance.

**Results:**

As data from the original MOSAIC trial showed no differences in cartilage volume and loss in the lateral compartment of the knee joint between the two treatment groups in any comparison, only the medial compartment and its subregions were studied. Stratification according to the median biomarker levels was used to discriminate treatment effect. In patients with levels of biomarkers of inflammation (HA, leptin and adipsin) lower than the median, those treated with chondroitin sulfate demonstrated less cartilage volume loss in the medial compartment, condyle, and plateau (*p* ≤ 0.047). In contrast, patients treated with chondroitin sulfate with higher levels of MMP-1 and MMP-3, biomarkers of cartilage catabolism, had less cartilage volume loss in the medial compartment, condyle, and plateau (*p* ≤ 0.050). Patients with higher levels of PIIANP and CTX-1, biomarkers related to collagen anabolism and bone catabolism, respectively, had reduced cartilage volume loss in the medial condyle (*p* ≤ 0.026) in the chondroitin sulfate group.

**Conclusion:**

This study is suggestive of a potentially greater response to chondroitin sulfate treatment on cartilage volume loss in patients with knee OA with low level of inflammation and/or greater level of cartilage catabolism.

**Trial registration:**

This is a post hoc study. Original trial registration: ClinicalTrials.gov, NCT01354145. Registered on 13 May 2011.

**Electronic supplementary material:**

The online version of this article (doi:10.1186/s13075-017-1377-y) contains supplementary material, which is available to authorized users.

## Background

Osteoarthritis (OA) is one of the most highly prevalent joint diseases characterized by progressive articular structural changes and cartilage degradation. This disease represents an increasing burden from a medical, social and economic point of view, as it affects the majority of people in the second half of their lifespan, having a significant negative impact on quality of life. The primary etiological factors may be numerous; hence, in the majority of patients the disease is considered to be idiopathic. At present, pharmacologic therapies only reduce symptoms, and they have adverse events. For this reason, attention has been focused on the investigation and development of new types of drugs that in addition to improving the clinical symptoms of OA and having better safety profiles can also decrease cartilage volume loss.

One such product is chondroitin sulfate, which is a sulfated glycosaminoglycan that has been shown to play a role not only in the metabolism of the proteoglycans (a major macromolecule in the cartilage), but also in OA joint tissues and cells, to improve the anabolic/catabolic balance of the extracellular cartilage matrix, and to reduce some pro-inflammatory and catabolic factors and the resorptive properties of subchondral bone osteoblasts [1–5]. In addition to its ability to reduce OA symptoms, similar to that of non-steroidal anti-inflammatory drugs (NSAIDs) [6, 7], in clinical trials and studies [6, 8, 9] it has also been shown to decrease cartilage volume loss as assessed by quantitative magnetic resonance imaging (qMRI). However, as mentioned in the original trial publication [6], although it has been recommended in some guidelines [10, 11], a general consensus has not been reached on the use of chondroitin sulfate [12, 13], due in part to the unavailability of prescription-quality products, indicated for use in OA, which have been evaluated by the US Food and Drug Administration (FDA) [12]. Over the years, several studies have looked into identifying biomarkers with predictive value to assess the risk of OA progression, particularly in patients with knee OA (see reviews [14–18]). A number of such biomarkers have been identified, predominantly in observational patient cohort studies, yet none has qualified for use in clinical practice for the follow up of patients with knee OA [14, 19]. There is an obvious need to identify prognostic biomarkers, but an even greater need to identify biomarkers that could predict patient response to potential disease-modifying OA drug treatments [14, 16, 20, 21].

Recently, the Osteoarthritis Research Society International-FDA (OARSI-FDA) Biomarkers Group identified twelve distinct biochemical biomarkers of bone and cartilage turnover that could be of interest and included in the BIPEDS classification system [17]. This group further looked at the predictive validity of eighteen biomarkers as OA-related predictors of radiographic and persistent pain progression [18]. They identified nine such biomarkers that were individually able to predict clinically relevant progression over 48 months; eight catabolic and one anabolic biomarker.

Most studies assessing biomarkers in relation to articular changes have used radiographic parameters, a technology that is not sensitive for the validation of biomarkers [14, 19, 20]. Converging data argue for the use of qMRI, another imaging technology that may assist in delineating the predictive value of biomarkers with regard to OA joint structural changes [14, 16, 22]. A few trials have used this technology to identify biomarkers that would be of value in predicting drug efficacy in cartilage volume loss and clinical outcomes such as total knee replacement [23, 24].

The present study aimed to explore whether biomarkers could identify patients with knee OA most likely to benefit from a chondroitin sulfate treatment, a commonly used treatment for knee OA. The information gathered from the present study could be very useful in the context of personalized medicine, as targeting responders would reduce the cost of treatment for OA patients and, importantly, improve patient benefits.

Using the knee OA patient cohort from a phase III clinical trial [6], eight serum biomarkers (C reactive protein (CRP), hyaluronic acid (HA), leptin, adipsin, matrix metalloproteinase-1 (MMP-1), MMP-3, N-terminal propeptide of collagen IIα (PIIANP) and C-terminal crosslinked telopeptide of type I collagen (CTX-1)) were assessed for their predictive value in determining the evolution of changes in cartilage in OA over a 2-year period by qMRI, and in relation to the effect of chondroitin sulfate treatment on cartilage volume loss. The rationale behind the selection of biomarkers was to use those that are representative of the most important pathways related to OA progression: inflammation (CRP, HA, leptin, and adipsin), cartilage catabolism (MMP-1, MMP-3) and anabolism (PIIANP), and bone remodeling (CTX-1), which have been found promising [16–18, 23, 24]. We reasoned that the biomarkers chosen for the study covered a broad range of pathways including the major pathological pathways of OA and were therefore likely to provide new information about biomarkers that could be associated with a treatment effect on cartilage volume loss.

## Methods

### Patients and treatments

This post hoc study used the patient cohort from a 2-year phase-III comparative, double-blind clinical trial (ClinicalTrials.gov Identifier NCT01354145) [6]. In brief, patients with symptomatic knee OA, diagnosed according to the clinical and radiological criteria of the American College of Rheumatology [25], and clinical synovitis, were randomized to explore the effects of chondroitin sulfate (Condrosan®; Bioibérica S.A.U., Barcelona, Spain) (1200 mg daily; *n* = 97) compared to celecoxib (200 mg daily; *n* = 97) on cartilage volume loss in knee OA. Celecoxib was chosen as the comparator since, among many other reasons, it was shown to have no effect on the progression of cartilage volume loss in patients with knee OA [26].

The original study protocol was approved by a central review board (Institutional Review Board (IRB) Services, Toronto, ON, Canada) and the IRB of the Centre hospitalier de l’Université de Sherbrooke (CHUS), Sherbrooke, QC, Canada. The original trial [6] was conducted in compliance with the ethical principles that have their origin in the Declaration of Helsinki (2000) and are consistent with “Good Clinical Practice” International Conference of Harmonization (ICH) Tripartite Guideline (January 1997) and the applicable laws and regulations of Canada, whichever afforded the greater protection to the individual. Ethical approval for this post hoc study was obtained with the original study, thus further approval was not required. All patients provided informed consent.

The according-to-protocol (ATP) population (chondroitin sulfate *n* = 57; celecoxib *n* = 62) was chosen as it included patients who fully complied with the study’s 2-year protocol and for whom serum and MRI at baseline and at 2 years were available. Of note, compared to the original ATP population [6], one serum sample was missing in the celecoxib group. These patients had taken the study medication throughout the entire study period (compliance ≥75%) and had no major protocol violations.

### Outcome

The primary outcome of the study was the usefulness of some serum biomarkers to indicate patients who are most likely to have reduction in cartilage volume loss in response to chondroitin sulfate. Of note, pain was not considered as an outcome because in the original MOSAIC trial [6] both therapeutic groups experienced a comparable reduction in disease-associated pain at 2 years. Moreover, data from the original trial [6] showed no difference between the two treatment groups in cartilage volume in the lateral compartment of the knee at baseline or loss of cartilage volume in any comparison; therefore, only the medial compartment of the knee (condyle plus plateau) and its sub-regions (condyle and plateau) were studied.

### Clinical, structural and biomarker assessments

#### Clinical evaluation at baseline

Clinical evaluation at baseline is shown in Table 1.

**Table 1:** Patient characteristics

	Chondroitin sulfate (*n* = 57)	Celecoxib (*n* = 62)	*P* value
Male, *n* (%)	26 (46)	29 (47)	0.899*
Age, years	61 ± 8	61 ± 8	0.909
Body mass index, kg/m^2^	30.3 ± 6.42	32.5 ± 5.9	*0.020*
Synovial membrane thickness, mm	1.04 ± 0.23	1.01 ± 0.22	0.528
Bone marrow lesion, score	2.49 ± 3.20	2.63 ± 2.33	0.291
Synovial fluid volume, mL	14.8 ± 15.9	11.2 ± 12.3	0.210
Joint swelling and effusion, *n* (%)	37 (65)	32 (52)	0.142*
Pain VAS, mm	60.6 ± 15.9	57.2 ± 18.6	0.252
WOMAC			
Total score (0–240)	121 ± 38	120 ± 46	0.934
Pain score (0–50)	24 ± 8	24 ± 9	0.909
Stiffness score (0–20)	11 ± 4	11 ± 4	0.694
Physical function score (0–170)	86 ± 29	85 ± 34	0.842
Quality of life (SF-36)			
Physical component summary	36.31 ± 7.82	36.33 ± 8.13	0.610
Cartilage volume (mm^3^)			
Medial			
Compartment	4586 ± 1517	4324 ± 1309	0.445
Condyle	2881 ± 1020	2682 ± 833	0.448
Plateau	1705 ± 545	1642 ± 532	0.529

Data shown are number of patients (%) or mean ± SD. Continuous variables were compared using the Student's *t* test/Mann-Whitney test; *p* values in italics are statistically significant. *Proportions were compared using the chi-squared test/Fisher’s exact test. *VAS* visual analog scale, *WOMAC* Western Ontario and McMaster Universities Osteoarthritis Index, *SF-36* Short Form 36

Clinical evaluation at baseline included assessment of knee pain on a visual analog scale (VAS), the Western Ontario and McMaster Universities Osteoarthritis Index (WOMAC) scores, the quality of life Short Form 36 General Health (SF-36), and clinical examination (swelling, visual examination; effusion, bulge sign).

#### MRI evaluation at baseline

Assessment using MR images included, in addition to cartilage volume [27, 28] (see subsequent description), the extent of synovitis assessed by measuring synovial thickness (mm) in four regions of interest (ROIs): the medial and lateral articular recess and the medial and lateral outer wall of the suprapatellar bursa [29]. The synovial fluid was determined using a fully automated system as previously described [30]. Bone marrow lesions were assessed in the same MRI sequences used for the cartilage assessment [31], and their extent was evaluated using the following scale: 0, absence; 1, <25%; 2, 25–50%; 3, >50% of the surface. The readers were blinded to treatment and to MRI examination time point except for baseline [6].

#### Cartilage volume assessment

Cartilage volume was assessed by MRI, which was performed on 1.5 T scanners (Siemens, Erlangen, Germany; General Electric, Milwaukee, WI, USA) using a standard knee coil, and the sequence acquisitions were as previously described [32]. The cartilage volume was measured by two experienced readers trained by musculoskeletal radiologists using the computer program Cartiscope™ (ArthroLab, Montreal, QC, Canada) as previously described [27, 28]. The change (percentage) in knee cartilage volume was obtained by subtracting the follow-up volume from the initial (baseline) volume divided by the initial (baseline) volume multiplied by 100. The percent coefficient of variation (CV%) is excellent as described [27].

#### Biomarker assessment

Blood samples were obtained after subjects fasted overnight. The samples were first allowed to coagulate and were then centrifuged (2200 rpm/1000 g, 10 minutes). Samples were stored at –80 °C until analyzed.

CTX-1 (Neo Scientific, Cambridge, MA, USA), HA (R&D Systems, Minneapolis, MN, USA) and PIIANP (EMD Millipore Corporation, Billerica, MA, USA) were assessed using specific ELISAs. Each assay is highly sensitive (sensitivity for CTX-1, 0.1 ng/mL; HA, 0.027 ng/mL; and PIIANP, 30.0 ng/mL) and designed to eliminate interference by other factors present in biological samples. Factors were read with a spectrophotometer (Multiskan Spectrum; Thermo Fisher Scientific, Mississauga, ON, Canada) and analyses performed with SkanIt sofware (Thermo). The CRP data (absolute values) available from the clinical trial report were used. The levels of the adipokines leptin and adipsin, and of MMP-1 and MMP-3 were assessed using the Human Magnetic Luminex Performance Assay kits (R&D Systems) in accordance with the manufacturer’s instructions with the minimum detectable dose (MDD) of 7.69 ng/mL; 1.8 pg/mL; 0.57 pg/mL and 1.8 pg/mL, respectively. The assessment was performed using a multiplex immunoassay (Luminex Corporation, Austin, TX, USA) on a LiquiChip 200. Analyte-specific antibodies were read using a Luminex analyzer, which discriminates each different analyte, and quantitated using LiquidChip Analyzer (QIAGEN, Toronto, ON, Canada). For each factor, an 8-point standard curve and appropriate controls were included, and samples were done in duplicate.

### Statistical analyses

Descriptive variables at baseline were presented as number, percentage, or mean ± standard deviation (SD) (Table 1). Differences between the two treatment groups were assessed using Student’s *t* test or the Mann-Whitney test for continuous variables with a non-normal distribution, and the chi-squared or Fisher’s exact test for categorical variables (Tables 1 and 2). Correlation between all biomarker levels at baseline were carried out using Pearson’s correlation analysis in all subjects (Table 3).

**Table 2:** Biomarker levels at baseline

	Chondroitin sulfate (*n* = 57)	Celecoxib (*n* = 62)	*P* value*
CRP, μg/mL^†^	4.02 ± 5.41 (*n* = 48)	5.56 ± 5.54 (*n* = 46)	*0.042*
HA, ng/mL	62.46 ± 44.81	72.46 ± 60.45	0.267
Leptin, ng/mL	37.60 ± 43.78	44.23 ± 38.53	0.214
Adipsin, μg/mL	4.05 ± 1.07	4.19 ± 1.07	0.638
MMP-1, ng/mL	5.11 ± 4.13	4.69 ± 3.15	0.827
MMP-3, ng/mL	13.03 ± 6.31	13.85 ± 11.04	0.720
PIIANP, μg/mL	4.10 ± 1.92	4.10 ± 1.61	0.468
CTX-1, ng/mL	0.59 ± 0.18	0.53 ± 0.12	*0.035*

Data shown are mean ± SD. *CRP* C reactive protein, *HA* hyaluronic acid, *MMP* matrix metalloproteinase, *PIIANP* N-terminal propeptide of collagen IIα, *CTX-*1 C-terminal crosslinked telopeptide of type I collagen. *Mann-Whitney test; *p* values in italics are statistically significant. ^†^Data missing for 25 patients at baseline

**Table 3:** Correlations between the biomarker levels at baseline in all subjects

		CRP (*n* = 94*)	HA (*n* = 119)	Leptin (*n* = 119)	Adipsin (*n* = 119)	MMP-1 (*n* = 119)	MMP-3 (*n* = 119)	PIIANP (*n* = 119)	CTX-1 (*n* = 117**)
CRP	*r*	-	-	-	-	-	-	-	-
	*p* value^†^	-	-	-	-	**-**	-	-	-
HA	*r*	<0.0001	-	-	-	-	-	-	-
	*p* value	>0.999	-	-	-	-	**-**	-	**-**
Leptin	*r*	0.344	-0.006	-	-	-	-	-	-
	*p* value	*0.001*	0.947	-	-	-	-	**-**	-
Adipsin	*r*	-0.016	0.188	0.343	-	-	-	-	-
	*p* value	0.881	*0.040*	*0.001*	-	-	-	-	-
MMP-1	*r*	0.045	-0.009	0.036	0.234	-	-	-	-
	*p* value	0.664	0.922	0.702	*0.010*	-	**-**	-	**-**
MMP-3	*r*	0.051	0.195	-0.245	0.049	-0.064	-	-	-
	*p* value	0.627	*0.033*	*0.007*	0.594	0.489	-	**-**	-
PIIANP	*r*	0.025	-0.042	0.015	-0.059	-0.277	-0.070	-	-
	*p* value	0.813	0.650	0.875	0.524	*0.002*	0.451	-	-
CTX-1	*r*	-0.093	0.063	0.016	-0.105	-0.049	-0.099	0.075	-
	*p* value	0.380	0.497	0.862	0.262	0.601	0.287	0.422	-

*CRP* C reactive protein, *HA* hyaluronic acid, *MMP* matrix metalloproteinase, *PIIANP* N-terminal propeptide of collagen IIα, *CTX-*1 C-terminal crosslinked telopeptide of type I collagen. *Data missing for 25 patients at baseline; **data missing for 2 patients at baseline

^†^Pearson’s correlation; *p * values in italics are statistically significant

Linear regressions adjusting for age, gender and body mass index (BMI) were also carried out to assess associations between baseline serum levels of the biomarkers and cartilage volume at baseline, and volume loss over 2 years within the medial compartment, medial condyle and medial plateau in all subjects (Table 4).

**Table 4:** Associations between cartilage volume and its loss and biomarker levels at baseline for all subjects

		CRP (*n* = 94*)	HA (*n* = 119)	Leptin (*n* = 119)	Adipsin (*n* = 119)	MMP-1 (*n* = 119)	MMP-3 (*n* = 119)	PIIANP (*n* = 119)	CTX-1 (*n* = 117**)
Cartilage volume at baseline									
Medial compartment	β	-49.24	-1.66	-4.26	101.26	-2.49	9.35	-40.65	-773.37
	*p* value^†^	*0.045*	0.409	0.385	0.315	0.931	0.454	0.489	0.256
Medial condyle	β	-37.09	-0.75	-2.62	74.50	-1.99	7.41	-38.66	-407.44
	*p* value	*0.030*	0.591	0.441	0.287	0.920	0.392	0.342	0.389
Medial plateau	β	-12.15	-0.91	-1.63	26.77	-0.50	1.94	-1.99	-365.94
	*p* value	0.170	0.215	0.361	0.468	0.962	0.671	0.926	0.141
Cartilage volume loss at 2 years									
Medial compartment	β	-0.179	0.001	0.009	0.334	0.049	-0.022	-0.546	3.418
	*p* value	*0.029*	0.905	0.590	0.342	0.622	0.615	*0.007*	0.150
Medial condyle	β	-0.184	0.004	-0.012	0.275	0.137	-0.038	-0.606	2.792
	*p* value	*0.050*	0.643	0.560	0.501	0.235	0.455	*0.010*	0.313
Medial plateau	β	-0.164	-0.005	0.049	0.415	-0.100	-0.001	-0.420	4.532
	*p* value	0.102	0.512	*0.016*	0.321	0.402	0.989	0.083	0.106

*CRP* C reactive protein, *HA* hyaluronic acid, *MMP* matrix metalloproteinase, *PIIANP* N-terminal propeptide of collagen IIα, *CTX-*1 C-terminal crosslinked telopeptide of type I collagen, *β* regression parameter estimate of the biomarker. *Data missing for 25 patients at baseline; **data missing for 2 patients at baseline. ^†^Linear regression adjusted for age, gender and body mass index; *p * values in italics are statistically significant

Since serum biomarkers are relatively new to the OA field in the context of a clinical trial, the median baseline biomarker levels were used to discriminate for the overall cohort (Additional file 1: Table S1) and within the treatment groups (chondroitin sulfate vs. celecoxib) (Table 5, Additional file 1: Table S2) to assess associations with baseline cartilage volume (medial compartment, medial condyle and medial plateau) and the predictive power of change in cartilage volume in the same regions at 2 years. The difference between mean cartilage volume within the three knee areas for lower vs. higher median biomarker levels was assessed by univariate analysis using Student’s *t* test or the Mann-Whitney test if the distribution was non-normal distribution (Additional file 1: Table S2).

**Table 5:** Two-year medial region cartilage volume loss (%) according to median value of baseline biomarker levels per treatment group

	Lower than median			Higher than median		
	Chondroitin Sulfate	Celecoxib	*P* value^†^	Chondroitin Sulfate	Celecoxib	*P* value^†^
CRP^a^ (*n* = 94)* Median 3.2 μg/mL						
*n*	28	19		20	27	
Medial compartment	-6.00 ± 3.31	-7.40 ± 3.81	0.237	-7.21 ± 3.49	-8.63 ± 4.65	0.510
Medial condyle	-5.13 ± 4.05	-6.99 ± 4.46	0.188	-6.05 ± 4.22	-8.28 ± 4.90	0.211
Medial plateau	-7.40 ± 3.90	-7.91 ± 4.49	0.745	-9.10 ± 5.12	-9.14 ± 5.56	0.642
HA^a^ (*n* = 119) Median 50.2 ng/mL						
*n*	31	28		34	26	
Medial compartment	-5.85 ± 3.24	-8.90 ± 3.95	0.083	-7.45 ± 3.11	-8.10 ± 4.46	0.629
Medial condyle	-4.99 ± 4.03	-8.57 ± 4.79	*0.047*	-6.61 ± 3.89	-7.73 ± 4.74	0.334
Medial clateau	-7.12 ± 3.74	-9.32 ± 4.32	0.275	-8.98 ± 4.50	-8.71 ± 5.33	0.671
LEPTIN^b^ (*n* = 119) Median 24.9 ng/ml						
*n*	30	30		27	32	
Medial compartment	-6.58 ± 3.12	-9.42 ± 4.50	*0.011*	-6.58 ± 3.45	-7.61 ± 3.89	0.445
Medial condyle	-5.90 ± 3.93	-9.16 ± 5.40	*0.014*	-5.54 ± 4.17	-7.16 ± 3.99	0.227
Medial plateau	-7.64 ± 3.08	-9.90 ± 4.90	*0.037*	-8.34 ± 5.17	-8.20 ± 4.85	0.846
ADIPSIN^b^ (n = 119) Median 4.1 μg/mL						
*n*	31	29		26	33	
Medial compartment	-6.48 ± 3.06	-8.76 ± 4.40	*0.040*	-6.70 ± 3.53	-8.20 ± 4.11	0.161
Medial condyle	-5.16 ± 3.83	-8.38 ± 4.97	*0.007*	-6.42 ± 4.19	-7.88 ± 4.60	0.249
Medial plateau	-8.64 ± 4.14	-9.35 ± 5.21	0.701	-7.16 ± 4.15	-8.67 ± 4.61	0.190
MMP-1^c^ (*n* = 119) Median 3.6 ng/mL						
*n*	25	34		32	28	
Medial compartment	-7.12 ± 3.38	-8.44 ± 3.68	0.239	-6.16 ± 3.14	-8.49 ± 4.86	*0.050*
Medial condyle	-6.29 ± 4.47	-8.20 ± 4.41	0.165	-5.30 ± 3.63	-8.01 ± 5.20	*0.028*
Medial plateau	-8.53 ± 3.48	-8.77 ± 4.46	0.908	-7.53 ± 4.65	-9.26 ± 5.40	0.269
MMP-3^c^ (*n* = 119) Median 11.2 ng/mL						
*n*	27	32		30	30	
Medial compartment	-7.10 ± 3.26	-7.29 ± 4.26	0.945	-6.12 ± 3.23	-9.72 ± 3.86	*0.001*
Medial condyle	-6.39 ± 3.81	-6.94 ± 4.45	0.765	-5.15 ± 4.16	-9.37 ± 4.79	*0.001*
Medial plateau	-8.21 ± 4.53	-7.67 ± 5.04	0.736	-7.75 ± 3.89	-10.40 ± 4.34	*0.048*
PIIANP^d^ (*n* = 119) Median 3.9 μg/mL						
*n*	28	31		29	31	
Medial compartment	-5.59 ± 2.96	-7.45 ± 4.02	0.115	-7.54 ± 3.28	-9.48 ± 4.23	0.070
Medial condyle	-4.91 ± 4.00	-6.97 ± 4.43	0.164	-6.52 ± 3.93	-9.25 ± 4.84	*0.026*
Medial plateau	-6.52 ± 3.23	-8.27 ± 5.04	0.129	-9.37 ± 4.54	-9.71 ± 4.67	0.829
CTX-1^d^ (*n* = 117)** Median 0.53 ng/mL						
*n*	23	35		33	26	
Medial compartment	-7.11 ± 3.46	-8.83 ± 4.14	0.170	-6.15 ± 3.13	-7.83 ± 4.37	0.085
Medial condyle	-6.43 ± 4.08	-8.38 ± 4.65	0.186	-5.20 ± 4.01	-7.67 ± 5.00	*0.025*
Medial plateau	-8.03 ± 4.14	-9.56 ± 4.78	0.237	-7.87 ± 4.29	-7.99 ± 4.89	0.931

Data are cartilage volume loss (%) expressed as mean ± SD. *CRP* C reactive protein, *HA* hyaluronic acid, *MMP* matrix metalloproteinase, *PIIANP* N-terminal propeptide of collagen IIα, *CTX-*1 C-terminal crosslinked telopeptide of type I collagen. Biomarkers related to ^a^inflammation; ^b^adipokines; ^c^matrix metalloproteinases; ^d^collagen metabolism. ^†^Analysis of covariance adjusted for age, gender, body mass index and, for HA only, cartilage volume at baseline; *p* values in italics are statistically significant. *Data missing for 25 patients at baseline; **data missing for 2 patients at baseline

Multivariate analyses (ANCOVA) were performed adjusting for the potential confounding factors at the onset of the study, including age, gender, BMI, and baseline cartilage volume for HA only (Table 5), as it is the only biomarker that showed, in patients with levels lower than the median, a statistically significant difference in the medial condyle between treatment groups (see Additional file 1: Table S2).

All tests were two-sided and a *p* value <0.05 was considered statistically significant. Since this was an exploratory study, no statistical corrections were made for multiple comparisons. All statistical analyses were performed using SAS software, version 9.3 (SAS Institute, Cary, NC, USA).

## Results

### Baseline demographic, clinical, and imaging data

The two therapeutic groups were balanced with the exception of BMI, which was slightly higher in the celecoxib group than in the chondroitin sulfate group (Table 1).

### Biomarkers

#### Baseline levels

In the study population, with the exception of lower values of CRP and higher values of CTX-1 in the chondroitin sulfate group, there was no significant difference between the two groups (Table 2). The differences in the levels of CTX-1, although statistically significant, were very small and unlikely to be clinically relevant. Moreover, those differences found for CRP and CTX-1 no longer existed when the data were stratified by the median value (data not shown).

#### Correlations between biomarker levels

There was positive correlation between levels (Table 3) of CRP and leptin, HA and adipsin, HA and MMP-3, leptin and adipsin, and adipsin and MMP-1. There was negative correlation between MMP-1 and PIIANP, and between leptin and MMP-3.

#### Associations between biomarkers and cartilage volume

A statistically significant negative adjusted (age, gender, BMI) linear association was found between the level of CRP and cartilage volume in the medial compartment and condyle at baseline (*p* = 0.045 and *p* = 0.030, respectively) (Table 4). A statistically significant negative adjusted association was also found between the levels of CRP and PIIANP and cartilage volume loss: medial compartment (*p* = 0.029 and *p* = 0.007, respectively) and condyle (*p* = 0.050 and *p* = 0.010, respectively). A statistically significant positive adjusted association was found between the level of leptin and cartilage volume loss in the plateau (*p* = 0.016).

#### Biomarker and cartilage volume levels at baseline and treatment effects

Since this work is among the first attempts to assess the impact of the studied biomarkers on knee OA structure as assessed by qMRI in relation to OA treatment, and no a priori cutoff values were available, we elected, as mentioned in “Statistical analysis”, to stratify the biomarkers at baseline according to their median levels. First, there were no differences in the cartilage volume between the two stratifications for any of the biomarkers (Additional file 1: Table S1). There were also no significant differences between the two treatment groups for each biomarker with the exception of HA, which, at baseline levels lower than the median, showed a statistically significant increase (*p* = 0.030) in volume for chondroitin sulfate vs. celecoxib (Additional file 1: Table S2).

Of all of the biomarkers studied (Table 5), only CRP did not show any difference in the extent of cartilage volume loss between the treatment groups, in either the lower or higher median group. The levels of all the other biomarkers were able to discriminate the extent of cartilage degradation between the treatment groups.

With regard to the biomarkers of inflammation (Table 5), compared to celecoxib, patients with levels of HA lower than the median treated with chondroitin sulfate demonstrated less cartilage volume loss in the medial condyle (*p* = 0.047), with a numerical trend toward significance in the compartment (*p* = 0.083).

Similarly, the lower levels of the two studied adipokines (Table 5), factors involved in inflammation and in cartilage catabolism, showed differentiation between the two treatment groups, with patients treated with chondroitin sulfate having less cartilage volume loss than with celecoxib. This was found in all medial sub-regions for leptin (*p* ≤ 0.037), and in the compartment (*p* = 0.040) and condyle (*p* = 0.007) for adipsin.

In contrast, for MMP-1 and MMP-3 (Table 5), it was in patients with the higher levels that differences between the two treatment groups were found: for MMP-1, patients treated with chondroitin sulfate had less cartilage volume loss than with celecoxib in the medial compartment (*p* = 0.050) and condyle (*p* = 0.028), and for MMP-3 in the medial compartment (*p* = 0.001) and both the condyle (*p* = 0.001) and plateau (*p* = 0.048).

For PIIANP and CTX-1 (Table 5), which are biomarkers related to collagen anabolism and catabolism, respectively, patients with higher baseline levels that were treated with chondroitin sulfate had less cartilage volume loss in the medial condyle compared to the celecoxib group (*p* ≤ 0.026).

## Discussion

Disease-modifying OA drug trials would benefit from the availability of biomarkers that could predict patients who are most likely to be responsive to a given treatment. The present study, although exploratory, provides important and novel information about the association of some serum biomarker levels with cartilage degradation, which could help to identify such patients. The original trial [6] demonstrated that chondroitin sulfate treatment in patients with knee OA had a significant beneficial effect on cartilage volume loss in the medial compartment (condyle). Here, we further showed that higher baseline values of the biomarkers PIIANP, CTX-1, MMP-1, and MMP-3, and lower baseline values of HA, leptin and adipsin seem to be associated with response to chondroitin sulfate treatment in reducing cartilage loss in patients with knee OA.

Data first showed that the extent of systemic inflammation at baseline, assessed by the levels of CRP and HA, positively correlated with the levels of cartilage catabolic factors such as leptin, adipsin, and MMP-3. Moreover, the level of leptin, an adipokine associated with inflammation, was found to positively correlate with the level of another adipokine, adipsin, which was also shown to be associated with cartilage degradation [24]. Interestingly, the inverse correlation between the levels of PIIANP and MMP-1 could likely reflect increased turnover and loss of type II collagen in cartilage, as PIIANP is associated with collagen formation/repair and MMP-1 with this macromolecule’s degradation. From these data, it can be hypothesized that joint inflammation often seen in knee OA and reflected by an increased level of CRP or HA, may induce an increase in the level of leptin and, secondarily, catabolic factors such as adipsin and MMPs [33], leading to cartilage degradation and loss, the latter reflected by negative correlation with PIIANP levels. Such a hypothesis is supported by the findings of this study, which show a negative association between the level of CRP at baseline and the cartilage volume at baseline, and cartilage loss at 2 years. This is well in line with a recent review on several related studies [34]. Our findings also concur with previous reports in which the level of CRP is correlated with disease progression in patients with knee OA assessed by X-rays and MRI, and an elevated level is associated with poorer disease outcome [23, 35–38]. However, these findings have not yet attained unanimity [39].

This study also showed that lower baseline values of leptin, adipsin and HA, and higher values of MMP-3 and MMP-1, were associated with greater reduction in cartilage volume loss with chondroitin sulfate than celecoxib. This suggests that patients with a milder level of inflammation, yet sufficient to induce the synthesis of cartilage catabolic factors (MMPs), and greater cartilage catabolism and disease progression [23], are more likely to be responsive to treatment with chondroitin sulfate. These findings are in line with those of previous studies [9, 40] that reported that OA patients who had a better response to glucosamine plus chondroitin sulfate treatment were those with less severe disease but greater cartilage degradation.

HA was found to be more strongly associated with the response to treatment with chondroitin sulfate than CRP. It could be speculated that HA may be more specific than CRP at assessing synovial inflammation. One might argue that the celecoxib treated patients had significantly higher CRP values at baseline and therefore a more severe degree of inflammation, which could have precluded a response to treatment. However, the stratification of patients based on the median value of CRP, which did not differ between treatment subgroups, does not provide support for such a hypothesis. HA has been found to be correlated with symptoms (WOMAC score) and with the degree of radiographic evidence of the disease (Kellgren-Lawrence (KL) grade) [41]. In the present study, patients with lower HA were more responsive to treatment with chondroitin sulfate, which, from the aforementioned findings and those in the review by Singh et al. [41], could mean that patients with less cartilage damage had a better response to this treatment.

The aforementioned data also agree with those from the adipokines, in that patients treated with chondroitin sulfate who had lower baseline levels of adipokines had less pronounced cartilage loss and likely less synovial inflammation [42], a finding that is in line with those related to HA. This has potential clinical relevance because leptin and adipsin have been associated not only with synovial inflammation but also with OA progression and cartilage degradation in both knee and hip OA as assessed by qMRI [24, 43, 44] and with the incidence of total knee replacement [24].

There was also evidence that the level of type II collagen synthesis in OA cartilage, assessed by measuring PIIANP, was clearly associated with response to treatment. The beneficial effect of chondroitin sulfate observed in patients with higher PIIANP values, which is usually indicative of less advanced disease [45], also supports the aforementioned assumption that the response to such treatment may be more pronounced in these patients.

The protective effect of chondroitin sulfate in patients with higher levels of CTX-I, reflecting an increase in the turnover of type I collagen in bone, is interesting and may possibly be related to the positive effect of chondroitin sulfate on bone marrow lesions [8] and on mechanisms related to the remodeling of bone in OA patients [2, 5, 46].

This study has limitations, the main one being related to the fact that the original study [6] was exploratory and therefore the analysis was performed on the available samples, which were from a limited number of patients. The absence of correction for multiple comparisons is also a study limitation, but should be taken in the context of an exploratory trial. Nevertheless, findings from this study agree with some [8, 47–54], but not all [55–58], previous trials/studies and meta-analyses. Pain could not be considered as a variable, as in the original MOSAIC trial [6] chondroitin sulfate showed a beneficial effect comparable to that of celecoxib. The data on CTX-1 were obtained from serum samples as, unfortunately, urine samples were not available. This could have had an impact on the correlation with other biomarkers. The absence of correlation between CRP and HA was somewhat surprising; the smaller number of samples for CRP could potentially have impacted the results of the analysis. Finally, the use of qMRI to assess the overall cartilage loss over time in knee OA provides a global appreciation of the change in knee cartilage volume in specific anatomical areas and represents a summation of cartilage loss (negative value) and areas of cartilage swelling (positive value), both of which are part of the OA cartilage changes observed during the evolution of knee OA.

## Conclusion

Although this study is exploratory and should be replicated with a larger number of patients to fully confirm the findings, data indicate that higher baseline values of the biomarkers related to collagen metabolism, PIIANP and CTX-1, and those related to cartilage degradation, MMP-1 and MMP-3, and lower baseline values of biomarkers related to inflammation, HA, leptin and adipsin, seem to be related to better response to chondroitin sulfate treatment in patients with knee OA. This is supportive of a potentially better response to treatment with chondroitin sulfate on cartilage volume loss in patients with knee OA with greater levels of cartilage catabolism and/or lower levels of inflammation.

## Additional file

Additional file 1: Table S1. Medial region cartilage volume at baseline according to median value of baseline biomarker levels. **Table S2.** Medial region cartilage volume at baseline according to median value of baseline biomarker levels per treatment group. (PDF 66 kb)

## Abbreviations

ANCOVA: Analysis of covariance
ATP: According-to-protocol
BMI: Body mass index
CRP: C reactive protein
CTX-1: C-terminal crosslinked telopeptide of type I collagen
ELISA: Enzyme-linked immunosorbent assay
HA: Hyaluronic acid
KL: Kellgren-Lawrence
MDD: Minimum detectable dose
MMP: Matrix metalloproteinase
NSAID: Non-steroidal anti-inflammatory drug
OA: Osteoarthritis
PIIANP: N-terminal propeptide of collagen IIα
qMRI: Quantitative magnetic resonance imaging
SD: Standard deviation
VAS: Visual analog scale
WOMAC: Western Ontario and McMaster Universities Osteoarthritis Index
