# Supplementary material for: Levels of serum biomarkers from a two-year multicentre trial are associated with treatment response on knee osteoarthritis cartilage loss as assessed by magnetic resonance imaging: an exploratory study
Source: Arthritis Res Ther. 2017 Jul 20;19:169. doi: 10.1186/s13075-017-1377-y (PMC5520291; doi:10.1186/s13075-017-1377-y)
Supplement: Additional file 1: Table S1. — Medial region cartilage volume at baseline according to median value of baseline biomarker levels. Table S2. Medial region cartilage volume at baseline according to median value of baseline biomarker levels per treatment group. (PDF 66 kb) [file 13075_2017_1377_MOESM1_ESM.pdf]

Additional file 1 – Tables

Table S1. Medial region cartilage volume at baseline according to median value of baseline biomarker levels

	Lower than median	Higher than median	p-value†
CRP[a] (n=94)*			
Median 3.2 µg/ml			
n	*47*	*47*	
Medial Compartment	4997±1588	4155±1215	0.182
Medial Condyle	3105±1077	2597±766	0.178
Medial Plateau	1892±566	1559±504	0.264
HA[a] (n=119)			
Median 50.2 ng/ml			
n	*59*	*60*	
Medial Compartment	4608±1520	4293±1292	>0.999
Medial Condyle	2873±1022	2682±824	0.995
Medial Plateau	1735±546	1610±526	0.990
LEPTIN[b] (n=119)			
Median 24.9 ng/ml			
n	*60*	*59*	
Medial Compartment	4944±1416	3946±1228	0.934
Medial Condyle	3069±560	2780±799	0.841
Medial Plateau	1874±516	1466±481	0.877
ADIPSIN[b] (n=119)			
Median 4.1 µg/ml			
n	*60*	*59*	
Medial Compartment	4454±1446	4444±1389	0.334
Medial Condyle	2765±941	2789±923	0.333
Medial Plateau	1688±572	1656±504	0.421
MMP-1[c] (n=119)			
Median 3.6 ng/ml			
n	*59*	*60*	
Medial Compartment	4532±1376	4368±1454	0.592
Medial Condyle	2827±871	2728±986	0.636
Medial Plateau	1705±552	1640±525	0.569
MMP-3[c] (n=119)			
Median 11.2 ng/ml			
n	*59*	*60*	
Medial Compartment	3998±1159	4893±1506	0.212
Medial Condyle	2498±793	3051±976	0.209
Medial Plateau	1500±414	1841±591	0.306
PIIANP[d] (n=119)			
Median 3.9 µg/ml			
n	*59*	*60*	
Medial Compartment	4586±1388	4315±1435	0.204
Medial Condyle	2882±905	2674±947	0.156
Medial Plateau	1704±541	1640±536	0.432
CTX-1[d] (n=117)**			
Median 0.53 ng/ml			
n	*58*	*59*	
Medial Compartment	4655±1588	4245±1218	0.376
Medial Condyle	2875±1052	2683±802	0.689
Medial Plateau	1780±595	1562±461	0.095

Biomarkers related to [a]inflammation; [b]adipokines; [c]matrix metalloproteinases; [d]collagen metabolism
Data shown are cartilage volume (mm^3) expressed as mean ± SD
†ANCOVA adjusted for age, gender and BMI
*Data missing for 25 patients at baseline; **data missing for 2 patients at baseline

Table S2. Medial region cartilage volume at baseline according to median value of baseline biomarker levels per treatment group

	Lower than median			Higher than median		
	Chondroitin Sulfate	Celecoxib	p-value†	Chondroitin Sulfate	Celecoxib	p-value†
CRP[a] (n=94)*						
Median 3.2 µg/ml						
n	*28*	*19*		*20*	*27*	
Medial Compartment	5165±1567	4749±1630	0.384	4117±1395	4184±1090	0.540
Medial Condyle	3238±1108	2910±1027	0.310	2600±869	2594±697	0.691
Medial Plateau	1927±519	1839±640	0.607	1516±556	1590±470	0.349
HA[a] (n=119)						
Median 50.2 ng/ml						
n	*31*	*28*		*34*	*26*	
Medial Compartment	5009±1672	4165±1211	**0.032**	4081±1145	4455±1388	0.300
Medial Condyle	3147±1141	2470±784	0.055	2563±758	2774±871	0.293
Medial Plateau	1862±585	1594±470	0.059	1517±433	1681±583	0.344
LEPTIN[b] (n=119)						
Median 24.9 ng/ml						
n	*30*	*30*		*27*	*32*	
Medial Compartment	5204±1530	4747±1237	0.214	3898±1188	3986±1278	0.600
Medial Condyle	3254±1055	2930±808	0.228	2466±811	2491±801	0.632
Medial Plateau	1951±528	1818±497	0.324	1432±427	1495±527	0.687
ADIPSIN[b] (n=119)						
Median 4.1 µg/ml						
n	*31*	*29*		*26*	*33*	
Medial Compartment	4527±1483	4376±1429	0.723	4655±1583	4278±1215	0.305
Medial Condyle	2840±1018	2686±862	0.690	2930±1040	2678±819	0.497
Medial Plateau	1687±521	1689±631	0.756	1726±582	1600±434	0.347
MMP-1[c] (n=119)						
Median 3.6 ng/ml						
n	*25*	*34*		*32*	*28*	
Medial Compartment	4760±1386	4364±1365	0.330	4449±1620	4275±1260	0.959
Medial Condyle	2966±908	2725±841	0.297	2814±1109	2629±834	0.750
Medial Plateau	1794±524	1639±570	0.211	1635±560	1645±493	0.943
MMP-3[c] (n=119)						
Median 11.2 ng/ml						
n	*27*	*32*		*30*	*30*	
Medial Compartment	4071±1320	3936±1020	0.982	5048±1553	4737±1466	0.428
Medial Condyle	2557±916	2448±683	0.909	3172±1034	2931±914	0.342
Medial Plateau	1515±447	1488±392	0.861	1876±575	1806±615	0.652
PIIANP[d] (n=119)						
Median 3.9 µg/ml						
n	*28*	*31*		*29*	*31*	
Medial Compartment	4621±1476	4554±1327	0.854	4551±1581	4094±1270	0.322
Medial Condyle	2897±1023	2867±802	0.909	2864±1034	2496±834	0.220
Medial Plateau	1724±518	1687±568	0.710	1687±579	1597±499	0.657
CTX-1[d] (n=117)**						
Median 0.53 ng/ml						
n	*23*	*35*		*33*	*26*	
Medial Compartment	4781±1767	4572±1479	0.763	4435±1349	4005±1003	0.348
Medial Condyle	3008±1229	2788±926	0.787	2787±871	2550±699	0.264
Medial Plateau	1772±597	1785±603	0.940	1648±972	1454±362	0.262

Biomarkers related to [a]inflammation; [b]adipokines; [c]matrix metalloproteinases; [d]collagen metabolism
Data shown are cartilage volume (mm^3) expressed as mean ± SD
†Student's t-test/Mann-Whitney test; bold indicates statistical significance
*Data missing for 25 patients at baseline; **data missing for 2 patients at baseline
